# Strabismus genetics across a spectrum of eye misalignment disorders

**DOI:** 10.1111/cge.12367

**Published:** 2014-01-01

**Authors:** XC Ye, V Pegado, MS Patel, WW Wasserman

**Affiliations:** a Centre for Molecular Medicine and Therapeutics, Child and Family Research Institute, Department of Medical GeneticsVancouver, BC, Canada; b Department of Ophthalmology and Visual Sciences, University of British ColumbiaVancouver, BC, Canada; c Child and Family Research Institute and Department of Medical Genetics, University of British ColumbiaVancouver, BC, Canada

**Keywords:** Duane retraction syndrome, genetics, linkage analysis, non-syndromic strabismus

## Abstract

Eye misalignment, called strabismus, is amongst the most common phenotypes observed, occurring in up to 5% of individuals in a studied population. While misalignment is frequently observed in rare complex syndromes, the majority of strabismus cases are non-syndromic. Over the past decade, genes and pathways associated with syndromic forms of strabismus have emerged, but the genes contributing to non-syndromic strabismus remain elusive. Genetic testing for strabismus risk may allow for earlier diagnosis and treatment, as well as decreased frequency of surgery. We review human and model organism literature describing non-syndromic strabismus, including family, twin, linkage, and gene expression studies. Recent advances in the genetics of Duane retraction syndrome are considered, as relatives of those impacted show elevated familial rates of non-syndromic strabismus. As whole genome sequencing efforts are advancing for the discovery of the elusive strabismus genes, this overview is intended to support the interpretation of the new findings.

## Conflict of interest

None of the authors has a conflict of interest related to any of the content of this review.

Strabismus (eye misalignment) is one of the earliest recorded genetic disorders. More than 2400 years ago, Hippocrates observed ‘Children of parents having distorted eyes squint also for the most part’. 1 Strabismus can cause visual problems during development, including loss of binocular vision, amblyopia (‘lazy eye’), and abnormal retinal correspondence (shifting of the fixation point relative to the macula in one eye). Strabismus disrupts stereopsis, which impacts the performance of numerous practical tasks requiring the precise judgment of distance (e.g. driving) or depth (e.g. microscopy) 2. In addition to reduced visual function, strabismus is associated with psychosocial problems impacting self-image, interpersonal relationships, performance in school and employment 3. Children as young as 5 years display a reduced tendency to interact with peers with noticeable strabismus 4,5. Strabismus negatively impacts employment rates and thus economic status 6. Strabismus surgery has positive impact on quality-adjusted life years (QALY), increasing QALY by 2.61, while being highly cost-effective ($1632/QALY) 7. While non-surgical intervention therapies (e.g. patching) in young children have not been similarly quantified, such practice is intended to reduce the need for surgical intervention.

The prevalence of strabismus is 2–4% among Caucasians, 2.4% among Hispanic/Latinos, 2.5% among African-Americans, and 1% in East-Asians 8–11. Among Caucasians, esotropia (inward misalignment) is three times more common than exotropia, while exotropia predominates in Cameroon black (63% of cases) and Asian populations (more than 70% of cases) 12–15. Studies consistently report balanced distribution between genders 16–19. In most cases, non-syndromic strabismus is characterized by non-restrictive, non-paralytic ocular misalignment with the same magnitude in all directions of gaze, which is known as concomitant (comitant) strabismus. Incomitant strabismus is paralytic in origin and the angle of deviation varies in different directions. The occurrence of muscle paralysis can be determined by the broad H test, which is scored positive if one eye lags behind the other in at least one of the six positions of gaze 20.

While the causes of non-syndromic strabismus are largely unknown, twin studies and family studies have demonstrated a substantial genetic contribution to strabismus 21. Although the heritability of strabismus has long been recognized, most advances at the level of specific genes have occurred during the past decade 8,12. Thus far, only a single non-syndromic strabismus locus on chromosome 7 has been confirmed to act in more than one family, and in those families the specific causal alterations have not been determined.

In this review, we summarize strabismus etiology and pathogenesis, genetic studies of non-syndromic strabismus and Duane retraction syndrome (DRS), as non-syndromic strabismus occurs at elevated rates in affected families 22, and describe model organism studies related to genetic forms of strabismus.

## Etiology and pathogenesis

**Figure 1:**
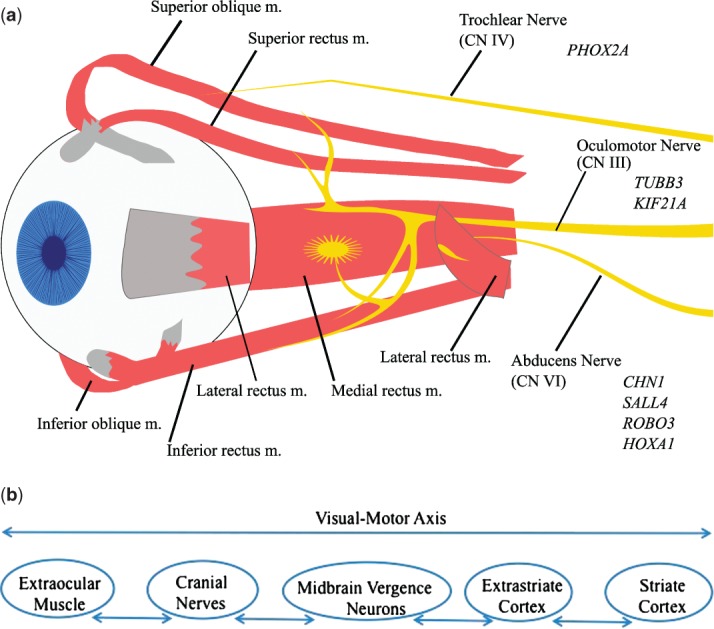
(a) A schematic representation of EOMs and nerve innervation with associated genes. CN, cranial nerve; m., muscle. (b) Defects along the visual-motor axis can contribute to infantile esotropia.

The mechanisms underlying strabismus may involve one of several systems or tissues (Fig. 1). Past reports highlight the potential for disruptions in extraocular muscles (EOM), orbital connective tissues, cranial nerves, fusion centers, and the visual cortex 23. The position of the eye is determined by all the five components. Mechanical trauma, acquired inflammation or infiltration, and metabolic disorder can all lead to EOM myopathy and secondary strabismus. Abnormalities of either the location or stability of the connective tissue pulleys alter the direction of EOM pulling and contribute to both congenital and acquired strabismus. Congenital cranial dysinnervation disorders (CCDDs) have been associated with hypoplastic or misrouted motor nerves to EOMs, and additional cranial nerve abnormalities have been observed 23. Fusion centers include a convergent center at the rostral–dorsal midbrain and a divergence center that, based on acute onset of concomitant esotropia related to tumors, is likely situated in the hindbrain 24,25. Animal experiments show that abnormal early visual experience can lead to strabismus and cause changes in metabolic activity in the visual cortex 26.

The age of onset distribution for strabismus is bimodal, with approximately 22% diagnosed before the age of 12 months and approximately 43% detected between 2 and 3 years of age. Non-accommodative strabismus was more common in the first group, while accommodative strabismus was more common in the second group (where accommodative refers to strabismus arising with altered visual acuity) 27. Approximately 26% of first-degree relatives of patients with hypermetropic (far-sighted) accommodative esotropia were affected with strabismus 28, suggesting that individuals with inherited hypermetropia may be predisposed to strabismus. However, a recent study demonstrated that heritability of strabismus was independent of refractive error. Bivariate analysis indicated a phenotypic correlation of only 0.20 between refractive error and eso-deviation, including tropia (constant eye misalignment) and phoria (latent eye misalignment); in other words genetic contributions to strabismus and hypermetropia are largely independent 29.

As indicated above, pathogenesis of infantile esotropia may result from defects spanning the visual-motor axis (Fig. 1b) 30. Researchers have postulated about the relationship between strabismus and changes in the visual cortex. At the turn of the 20th century, Worth proposed that infantile esotropia was due to an inborn defect of fusion, as surgery on EOM could not reverse strabismus 31. Tychsen suggested that this fusion faculty was situated within the striate cortex, and specifically proposing that congenital defects would therefore be present in disparity-sensitive, binocular neurons 30. Using staining techniques, a paucity of such binocular connections was observed in both natural and induced strabismic monkeys while monocular connections remained. Electrophysiological measurement showed that loss of binocular responsiveness and disparity sensitivity was consistent with the reduced number of binocular connections 32.

Hypotheses for strabismus mechanisms have been proposed which focus on the subcortical visual pathway, brainstem vergence motoneurons, the brainstem vestibule-ocular pathway, and cranial nerves 32. On the other end of the visual-motor axis, Chavasse proposed a ‘motor’ hypothesis, suggesting that abnormal optical input, such as weakness of the EOM, may impede development of binocular fusion thus leading to strabismus. He argued that surgery in the very young age to restore eye alignment could rescue binocular vision 32,33. Clinical data showed that shorter durations of misalignment correlated with better stereopsis, implying that muscle abnormalities lead to poor stereopsis, not vice versa 33. Examination of strabismic EOM identifies some abnormalities. A 2012 magnetic resonance imaging study of 12 concomitant esotropes and 13 controls demonstrated rectus muscle enlargement. Cross sections of medial rectus muscle were up to 39% larger (p < 0.005), and those of lateral rectus muscle were up to 28% larger in the esotropic cases. Moreover, medial rectus contractility was 60% higher in exotropic individuals (p < 0.005) 34. It is inconclusive, however, whether the structural changes in EOMs are the cause of strabismus or merely reflect the adaptation to the change of motoneuron firing patterns, as observed in other skeletal muscle tissue 35. Schoeff et al. reasoned that the lack of evidence of EOM denervation or dysinnervation in non-syndromic strabismus suggested a visual cortex contribution 34. As live imaging technology advances, higher resolution examination may advance our understanding of the relative contribution of defects in muscle and nerves to the strabismus phenotype.

## Risk factor

Significant strabismus risk factors include retinopathy of prematurity, low birth weight, premature birth, and smoking during pregnancy. As our focus will remain on genetic risk, the interested reader may find additional information about the other factors in the systematic review by Maconachie et al. 19.

## Family and twin studies

Many early studies focused on the transmission of strabismus through families. However, findings varied in terms of heritability, inheritance mode, and the concordance of strabismic types 19. Surveys conducted between 1910 and 1950 indicated that hereditary factors ranged from 20% to 50% in families with esotropia 36. Schlossman and Priestley found that 47.5% of 158 patients with strabismus, 48.9% of 139 esotropes, and 36.8% of 19 exotropes belonged to families with two or more additional affected members. The authors suggested that the actual number might be larger since subtle alignment deviations could be missed 37. The highest reported familial incidence of strabismus was 65% 16,28.

A longitudinal study found that 18% of 34 babies born in families with a parent affected by convergent (i.e. esotropia) strabismus developed constant or intermittent esotropia by 6 months 38. As the types of assessed relatives varied between studies and there was no consideration of environment, the precise genetic risk is unclear. Nevertheless, the figures were much higher than those in general population (approximately 5%), supporting a contribution of genetics to strabismus risk. The concordance of strabismus types varied across the studies. Families with a mixture of esotropia and exotropia phenotypes were reported 13,37. One study found that 80% of strabismus cases occurring in the same family were concordant 19. Another study reported 54% concordance within 39 studied families 13.

As familial clustering of strabismus can reflect either a common genetic factor or an unrecognized environmental factor, twin studies are the key to quantify the relative genetic contribution. Twin studies of strabismus have reported higher concordance rates in monozygotic twins than dizygotic twins, suggesting a predominant genetic factor 19. Matsuo et al.’s twin study showed that strabismic subtypes of 67.3% of 49 pairs or sets were concordant, and the concordance rate was higher in monozygosity (82.4%) than in multizygosity (47.6%) 39. Wilmer and Backus performed a meta analysis, reporting monozygosity and dizygosity concordances of 54% and 14%, respectively, in studies with systemic ascertainment; and 66% and 19%, respectively, without systematic ascertainment 40. This contradicted with Paul and Hardage’s 1994 study, but Wilmer and Backus observed that a translation error in the 1994 study led to an overestimation of dizygosity concordance 40,41. Podgor reported that the odds ratio for esotropia rose from 2.6 if a sibling from a preceding birth was affected to a ratio of 5.4 if a twin (or other multiple birth) was affected 21.

Esotropia and exotropia have a strikingly different genetic risk profile. In the Podgor study, a striking odds ratio of 330 was reported for exotropia in cases of multiple birth with one affected twin, while single births had an extremely low odds ratio of 2.2, data most consistent with a strong multiple birth environmental impact on exotropia risk 21. A study with 1462 twins suggested that genetic heritability was specific to esotropia, reporting that heritability of eso-deviation was 64% while no heritability was detected for exo-deviation 29. Exotropia (75%) had higher observed concordance than esotropia (65.7%) in a Chinese twin study, which may reflect influence of both the multiple birth environmental influence on esotropia and potential ethnic differences in the genetic contribution to esotropia 19,42.

A key consideration arises from twin studies. Wilmer and Backus raised the potential confounding contribution of phoria to the study of strabismus genetics. Phoria is a latent misalignment of the eyes that appears when fixation on a target is broken (which can be revealed with a cross-cover test). Wilmer and Backus observed that genetic factors were necessary for strabismus development but not for phoria development 40. Phoria cases have been noted in families with strabismus, and a portion of strabismus genetics studies have included phoria as positive cases 13.

Summarizing the above information, esotropia is most closely tied to heritable factors while exotropia has a stronger environmental component. Future studies should therefore be designed in a manner that controls for the environmental component, including multiple births.

## Genetic mechanisms

Dominant, recessive, and sex-linked inheritance patterns have been proposed for non-syndromic strabismus in family studies 19,37. In different families, Czellitzer reportedly suggested two recessive genes were responsible for strabismus, while Waardenburg proposed a model of a single autosomal gene 37,43. A study using quantitative measurement of sensory and motor function rejected the theories of Mendelian inheritance of strabismus as a single trait 14. The majority of studies have noted that simple Mendelian models cannot explain the complexity of strabismus inheritance patterns. There are multiple genetic mechanisms represented in the families described in the scientific literature. Furthermore, the high frequency of strabismus may confound family studies with some cases likely arising from environmental mechanisms. Without accurate categorization based on exquisite pathological characterization of the strabismus, and given the diversity of potential physical mechanisms, such conflicting results are not entirely unexpected.

## Linkage analysis

Parikh et al. identified the first concomitant strabismus locus on chromosome 7p22.1 (STBMS1) in a linkage analysis of a large family. Among seven initially assessed multiplex families with non-syndromic strabismus, one family showed a significant logarithm of the odds (LOD) score on chromosome 7. Although the pedigree suggested an autosomal dominant inheritance pattern, the haplotype data was most consistent with an autosomal recessive model or a more complex model, such as semi-dominant inheritance 44. The autosomal recessive inheritance model has been subject to discussion 12. The other six families in the original study were not consistent with the chromosome 7 loci contributing 44. In the subject family, eight of fourteen siblings were affected, and seven of these eight patients had hypermetropia of varying severity. Rice et al. examined 12 additional families, of which one was consistent with an STBMS1 role. Five affected family members had primary non-syndromic comitant esotropia while 21 examined family members were unaffected. In this second STBMS1 family, the pattern of inheritance best fits a dominant mode of inheritance 45. In combination the reports indicate that there is at least one non-syndromic strabismus associated genetic component at the STBMS1 locus. Elucidating the causal mutations in the two families may clarify the conflict between transmission models.

The Ohtsuki group tried to identify comitant strabismus susceptibility loci through sib-pair analysis and nonparametric linkage analysis for multiple pedigrees. This initial 2003 attempt indicated multiple loci with low LOD scores 46. A 2008 report identified 4q28.3 and 7q31.2 loci as having significant evidence of linkage. After stratifying cases into esotrpoia and exotropia subgroups, they identified additional loci at 8q24.21 and 14q21.3, respectively 47.

A summary of reported candidate loci for comitant strabismus is presented in Table1. Based on the range of findings, it appears likely that multiple genes are contributing to familial forms of strabismus. Elucidating the specific genes remains a grand challenge for the field, but emerging genome sequencing tools may generate a new wave of insights.

**Table 1:** Selected comitant strabismus associated loci

Loci	Inheritance pattern	Ethinicity	Phenotype	PMID
7p22.1(STBMS1)	Recessive	European	Esotropia in infancy or childhood, 7 of 8 affected individuals had various degree of hypermetropia	14519848 44
7p22.1(STBMS1)	Dominant	Northern Irish	Primary non-syndromic comitant esotropia	19218600 45
16p13.12-p12.3	Recessive	Saudi Arabian	Infantile esotropia and esotropic Duane retraction syndrome	21541264 93
4q28.3	Dominant	Japanese	Comitant strabismus	18824738 47
7q31.2	Recessive (Imprinting)	Japanese	Comitant strabismus	18824738 47 19597570 94
6q26	Imprinting	Japanese	Comitant strabismus	19597570 94
12q24.32	Imprinting	Japanese	Comitant strabismus	19597570 94
19q13.11	Imprinting	Japanese	Comitant strabismus	19597570 94

## Gene expression studies

Experimental approaches to elucidate molecular mechanisms related to strabismus have been pursued. Microarray analysis showed that expression of 604 genes differ significantly between 100 strabismic EOM samples and 28 normal EOM samples. Together with PCR experiments, three major conclusions were drawn. Collagen and collagen-related genes were upregulated; specific myosins, such as EOM-specific myosin (*MYH13*) and myosin heavy chain-1 (*MYH1*), and related contractile genes were downregulated; genes involved in energy balance, such as mitochondrion homeostasis or regulations of energy metabolism, were dysregulated in strabismic EOMs. The conclusions should be assessed with caution, since it was not specified which forms of strabismus were represented in the samples, although the authors suggested that the sample set may have a high portion of exotropia cases 48.

In another study, expression levels of seven myogenesis-related genes in EOMs from 18 concomitant strabismus patients were compared against 12 samples from a single non-strabismic individual. Six of the genes had reduced expression levels, leading Zhu et al. to suggest that altered growth of muscles may be involved. However, it was unclear whether the patients had congenital strabismus nor the nature of the deviations involved 49. Furthermore, the two sample sets were collected in distinct ways (i.e. obtained from corrective surgery *vs* cadavers), which has been recognized to cause difficulty in the interpretation of gene expression studies 50,51.

## Duane retraction syndrome

While the focus of this review is the genetics of non-syndromic forms of strabismus, there are familial syndromes in which strabismus rates are elevated in otherwise non-syndromic family members. About 70% of DRS cases do not exhibit other congenital abnormalities, and approximately 20% of cases have a family history of strabismus 22,52. Overall DRS accounts for approximately 5% of strabismus cases 53. DRS is a congenital cranial dysinnervation disorder. Based on these observations, we include DRS in this review as we perceive an opportunity to find common causal genes between non-syndromic strabismus and DRS.

Three types of DRS have been described based on clinical examination. In these studies, key attributes include abduction, movement of a body part away from the midline, and adduction, movement toward the midline. Type 1 DRS is characterized by marked limitation of abduction, type 2 DRS is characterized by marked limitation of adduction, and type 3 DRS is characterized by a combination of marked limitation of both 54. The majority (60%) of diagnosed DRS cases are female. Up to 60% of all cases are bilateral, and up to 80% of unilateral cases are left-sided 54,55. Wabbels et al. found predominant females cases (64%) and left eye involvement of unilateral cases (72%), whereas bilateral only accounted for 12% of cases 56.

While most cases are sporadic, reports of familial DRS date back to 1896 57. Up to 10% of Duane anomalies are inherited in an autosomal dominant fashion 58. The connection between infantile esotropia and DRS are illustrated by recent studies. In the Strabismus Inheritance Study in Tasmania (SIST), a set of 133 families with infantile esotropia was recruited, of which multiple members were affected with DRS in two families. A separate set of 40 families with at least one case of DRS were recruited, of which 21 had a familial history of ocular motility disorders but only two had multiple members affected by DRS 54. Linkage analysis had previously shown linkage between 8q12-13 and Duane syndrome. The SIST study confirmed a prior association of both DRS and infantile esotropia with partial trisomy 8 59,60. Combining this information, a gene-dosage mechanism was proposed 54. Separately, Khan et al. identified two susceptibility loci, 3p26.3-26.4 and 6q24.2-25.1 using multipoint linkage analysis in a consanguineous family with four affected children (one with DRS and three with non-syndromic esotropia) 22.

### Chromosome 8q and type 1 DRS

The focus on chromosome 8q in DRS studies has progressed to the search for a causal gene in the loci, but no clear single causal gene has been established. A *de novo* reciprocal balanced translocation t(6;8)(q26;q13) was identified in a patient with DRS. This patient had amblyopia and narrowing of palpebral fissures 61. The carboxypeptidase A6 (*CPA6*) gene at the previously identified DURS1 (DRS-1) locus on chromosome 8 was disrupted between the first two exons in this patient and was proposed as the causal gene 62. CPA6, a member of the M14 metallocarboxypeptidase family, is expressed in a limited number of tissues in mice, including the rectus muscle layer of the embryonic eye. In adult mouse, CPA6 was expressed in olfactory bulb and other parts of the brain 63. CPA6 knockdown using morpholino antisense oligos in zebrafish did not produce a phenotype, contradicting a dosage hypothesis 54,64. No pathogenic *CPA6* mutations were identified in a set of 18 sporadic DRS patients 61. Two patients with microduplication of 8q12 displayed multiple congenital anomalies, including DRS 65,66. Studying a third patient with similar phenotype, including DRS, a recent study identified the minimal critical region at the loci of 1.2 Mb, excluding *CPA6*. *CHD7* duplication was suggested to be responsible for at least part of the features in resulting from the 8q12 duplication 67. Reported duplications and deletions in affected individuals do not overlap, suggesting either multiple contributing genes or a gene with distal regulatory regions might be responsible 68. Although the chromosome region 8q12-q13 has been linked to DRS1 in multiple cases, more study is required before a definite conclusion can be drawn about the causal gene.

### *CHN1* and type 2 DRS

The *CHN1* gene has been more clearly demonstrated to be a causal gene for DRS2. *CHN1* is located on chromosome 2 and encodes two Rac-specific guanosine triphosphatase (GTPase)-activating alpha-2-chimerin isoforms. Miyake et al. identified seven heterozygous missense mutations in seven unrelated DRS2 families co-segregating with the affected haplotypes 69. These mutations were neither recorded in the single nucleotide polymorphism database nor observed on 788 control chromosomes. *CHN1* mutations were present in 7 of 20 (35%) examined DRS families, while no *CHN1* mutations were observed in 140 sporadic DRS patients 70. Predicted gain-of-function mutations in *CHN1* were found in two families with type 2 DRS 71. Overexpression of wild-type alpha-2-chimerin in the chick embryonic oculomotor nucleus led to stalling of oculomotor nerve growth and the premature axon termination adjacent to the dorsal rectus muscle, supporting a functional role for CHN1 in DRS 69.

### Type 3 DRS

While loci have been established that account for a portion of type 1 and type 2 DRS, the genetic components of type 3 DRS are more elusive. It is possible that the type 3 DRS is more heterogeneous than the other two classes. In a thin-sectioned magnetic resonance imaging (MRI) study, the abducens nerve was reliably observed in 60 eyes of 30 individuals from a control group. The abducens nerve on the affected eye was absent in 18 of 18 eyes from 16 patients with type 1 DRS, and in 2 of 2 eyes from type 2 DRS patients. The nerve was absent in only 3 of 5 eyes from five patients with type 3 DRS 72. The clinical heterogeneity in type 3 DRS may reflect genetic heterogeneity.

### Okihiro syndrome

In addition to the ocular anomalies of the basic form of DRS, Okihiro syndrome (also called Duane-radial ray syndrome) is associated with additional abnormalities affecting the upper limbs and, less commonly with renal anomalies and sensorineural hearing loss 73. Autopsy and MRI studies of Okihiro syndrome patients have revealed hypoplasia or absence of the sixth nerve nucleus (i.e. abducens nerve) on the affected side, the ipsilateral lateral rectus being innervated by branches of the oculomotor nerve 74,75.

Mutations in the *SALL4* zinc finger transcription factor gene were the first causal genetic alterations discovered for Okihiro syndrome patients 73. The discovery arose when Kohlhase et al. proposed that Okihiro syndrome might be due to mutations in a SALL gene family member based on phenotype overlap between Okihiro syndrome and Townes-Brocks syndrome, which is caused by mutations in the *SALL1* gene. They successfully identified mutations in *SALL4* gene from five of eight Okihiro families 76. Al-Baradie et al. identified a nonsense mutation in *SALL4* gene in affected individuals originally reported by Okihiro et al. in 1977, as well as 2 additional families 77. The broader DRS phenotype is present in approximately 70% of *SALL4* mutations carriers 78. A mouse model shows that Sall4 is regulated by Tbx5 transcription factor; both genes contribute to patterning and morphogenesis of the anterior forelimb and heart 79. This observation explains the shared endophenotypes between Okihiro syndrome and Holt–Oram syndrome, which is associated with mutations in the *TBX5* gene. Whole mount *in situ* hybridization analysis of Sall4 expression during mouse embryogenesis shows prominent expression in midbrain and branchial arches and suggests that a dosage reduction of Sall4 might disrupt abducens nerve development 78,79.

## Animal models

Although the genetic origins of strabismus remain to be fully deciphered, several animal models of the phenotype have been studied and may serve as resources in the search for causal genes. Most of the model animals described below are albinos, with pigmentation loss ranging from partial to complete. Visual abnormalities, including strabismus, have been linked with albinism in diverse mammals such as albino primates, white tigers, and albino cats (including Siamese cats) 80–83.

### Famous strabismic animals

Animals with cross-eyes have become popular images on the Internet. Joco, a cross-eyed lion at the Erfurt Zoo (Germany) is most likely to suffer from congenital strabismus. The cross-eyed opossum Heidi at the Leipzig Zoo (Germany) became a celebrity, but the condition was likely environmentally triggered. The causes of strabismus in animals vary, with only a portion deriving from genetic influence. Finding suitable animal models for the study of non-syndromic strabismus could accelerate research efforts.

### Cats

In Siamese cats, a temperature-sensitive mutated *TYR* gene encoding tyrosinase is expressed normally in cooler extremities, giving a darker color, while expression is reduced in warmer parts of the body, leading to poor pigmentation. Anatomical studies show that axons of temporal retinal ganglion cells go to the opposite side of the brain instead of staying on the same side as observed in non-albino cats 84,85. The misrouting defects are also observed in albino mice and rabbits with TYR defects. Insertion of functional *TYR* genes into such albino mice and rabbits corrects for axon misrouting 86. Humans with ocular albinism also show abnormal decussation (crossing) of optic neurons, causing reduced or absent binocularity. This characteristic is associated with elevated prevalence of strabismus 87. Nevertheless, there is not yet convincing evidence that *TYR* mutations contribute to strabismus in humans. While the link between strabismus and axon misrouting is unknown, genes directly involved in optic chiasm development might be considered as candidates 88.

The unusual axon wiring pattern observed in TYR defective albino animals raises concern that these animals may not be suitable models for human strabismus. Artificially induced strabismus models, such as those established by tenotomy (tendon lengthening) and by exposure to early abnormal visual experience, may be similarly ill-suited to study genetic influences on strabismus. To evaluate the relevance of artificially induced strabismic cats, the ocular dominance distributions for cats with induced strabismus and natural strabismus were compared and found to be similar. Approximately 35% of cells were monocular in either strabismus group, but a statistically significant difference was noted with normal cats, which have 81% binocular cells 89. Work with the animal models continues, exemplified by a study which showed that early induced unilateral convergent strabismus in cats led to abnormal corpus callosum connection 90. Such experiments highlight how abnormal early visual experience impacts visual cortex development, but do not provide a clear path for using induced animal models to track down key genes. Thus the study of non-syndromic strabismus could benefit from efforts to identify additional eye misalignment animal models.

## Conclusion and future directions

The causal genes predisposing to non-syndromic forms of strabismus remain to be discovered. The combination of next generation sequencing with both large-scale populations and targeted families may soon reveal critical genes and consequently confirm or expose critical molecular mechanisms. Genome-wide association studies have been reported to be underway, while exome sequencing family-specific studies of non-syndromic strabismus are likely to emerge soon 91,92. Aided by the background presented in this overview, the discovery of critical genes causing non-syndromic strabismus will allow earlier identification of individuals who are at high-risk and thus most likely to benefit from effective early intervention treatments.
